# Retrograde intrarenal surgery with intelligent pressure control: experience from a primary hospital in China

**DOI:** 10.1186/s12894-025-01910-8

**Published:** 2025-08-26

**Authors:** Xin Liu, Wangsheng Huo, Yanjun Wang, Hui Chai, Libin Chen, Hui Meng, Zhihong Gong, Hongze Zhang, Jian Lang, Zhantian An, Wei Wei

**Affiliations:** 1 Henan Provincial People’s Hospital, Zhengzhou University People’s Hospital, Zhengzhou, China; 2 Xinjiang Production and Construction Corps 13th division Red Star Hospital, Hami, China

**Keywords:** Retrograde intrarenal surgery, Intelligent pressure control, Stone free rate

## Abstract

**Objective:**

This study aimed to compare the clinical outcomes of conventional retrograde intrarenal surgery (RIRS) and RIRS with intelligent pressure control, as well as to identify factors influencing the stone-free rate (SFR).

**Materials and methods:**

We conducted a retrospective review of 101 patients treated with either conventional RIRS or RIRS with intelligent pressure control from September 2023 to September 2024. Clinical and stone-related parameters were collected for comparison between the two methods, and factors affecting SFR were examined using univariate and multivariate logistic regression analyses.

**Results:**

The SFR was significantly higher in the intelligent pressure control group compared to the conventional RIRS group (89.8% vs. 73.1%, *p* = 0.032). No statistically significant differences were found between the two groups regarding operative time or post-operative hospital stays. The incidence of post-operative fever was lower in the intelligent pressure control group than in the conventional RIRS group (2.0% vs. 9.8%), although this difference did not reach statistical significance (*p* = 0.205). Univariate analysis identified stone size, stone density, number, location, and surgical method as factors associated with SFR. Multivariate analysis further confirmed that stone size, density, and surgical method significantly impacted SFR.

**Conclusion:**

RIRS with intelligent pressure control significantly improves the stone-free rate compared to conventional RIRS, without increasing complications, hospitalization duration, or operative time. Additionally, stone size and density were influential factors for SFR.

**Supplementary Information:**

The online version contains supplementary material available at 10.1186/s12894-025-01910-8.

## Introduction

The prevalence of urolithiasis worldwide ranges from 1–20%[1]. In China, a nationwide cross-sectional study reported approximately 5.9% of adults were affected by kidney stones [1]. According to clinical guidelines [2] [3]patients with stone burden ≤ 20 mm could be treated with URS, while patients with stone burden>20 mm are recommended to undergo PCNL as first-line therapy. For patients with lower pole stones measuring 10–20 mm, either surgical approach may be selected.

Retrograde intrarenal surgery (RIRS) with intelligent pressure control was first reported by Song [4] in 2016. This technique, utilizing medical perfusion and suction systems to regulate renal pelvic pressure and assist in stone extraction, is applicable for patients with renal and upper ureteral stones [5]. Reports indicate that the stone-free rate (SFR) achieved with this method is satisfactory [5] [6].

Although the innovative technique has been implemented on in China, most related studies were primarily conducted in high-level medical centers rather than in primary hospitals in China and there is a lack of direct comparisons with conventional flexible ureteroscopy. Therefore, our study aims to fill this gap by evaluating the effectiveness and safety of RIRS with intelligent pressure control in comparison to conventional RIRS, while also exploring factors influencing the stone-free rate (SFR).

## Materials and methods

### Study design and ethics approval

This study (No.20 in 2024) was a retrospective, single-center comparative cohort study and approved by the Ethics Committee of Xinjiang Production and Construction Corps 13th division Red Star Hospital. Patients who were hospitalized and received RIRS in Xinjiang Production and Construction Corps 13th division Red Star Hospital between September 2023 and September 2024 were included. Two senior urologists (WH and YW) participated in this study, all of whom had performed more than 100 flexible ureteroscopy procedures prior to the study period. Each surgeon was proficient in both conventional and intelligent pressure-controlled RIRS, and procedures in both groups were evenly distributed among them. Based on the type of surgery received, patients were assigned to one of two groups: conventional RIRS (Group A) and RIRS with intelligent pressure control (Group B). The choice between conventional RIRS and RIRS with intelligent pressure control was based on equipment availability and surgeon preference at the time of surgery.

### Inclusion and exclusion criteria

The inclusion criteria were as follows: (a) single stone in the kidney or upper ureter; or unilateral multiple stones in the kidney or upper ureter; (b) the cumulative stone burden between 10 mm and 30 mm. The exclusion criteria were: (a) bilateral urinary calculi and simultaneous surgery; (b) absence of a preoperative CT scan; (c) absence of a non-contrast CT (NCCT) scan prior to ureteral stent removal after 30 days of the surgery to evaluate the stone-free rate (SFR); (d) absence of other study-required indicators. A priori sample size calculation was conducted using a significance level (α) of 0.05, 80% power, and a non-inferiority margin of 5%. Based on preliminary institutional data showing stone-free rates of 80% in the intelligent pressure control group versus 50% in the conventional RIRS group, a minimum of 48 patients per group was required. Our actual enrollment of 52 patients in Group A and 49 in Group B meets this requirement and ensures sufficient statistical power for evaluating the primary endpoint.

### Surgical techniques

#### Conventional RIRS

After general anesthesia, the patient was placed in the lithotomy position. A ureteroscopy was performed under the guidance of a hydrophilic safety wire to evaluate the feasibility of flexible ureteroscopy. Once RIRS was deemed feasible, the guidewire (Urovision, Germany) was retained in the renal pelvis and ureter to facilitate the insertion of the ureteral access sheath (UAS) (Innovex, China). For ureteral stones, these were repositioned into the renal pelvis for further treatment. A disposable flexible ureteroscope (Vathin, China)was then advanced through the UAS to the renal pelvis. Upon locating the stone, lithotripsy was performed using a Holmium laser (Quanta System S.p.a, Italy) under guidance of flexible ureteroscope with an energy setting of 0.6–1.0 J, frequency setting of 10–20 Hz, and a stone retrieval basket (Innovex, China) was employed when necessary. Before concluding the procedure, a thorough inspection was conducted, and a 6 F double J stent (Urovision, Germany) was placed.

#### RIRS with intelligent pressure control

**Fig. 1:**
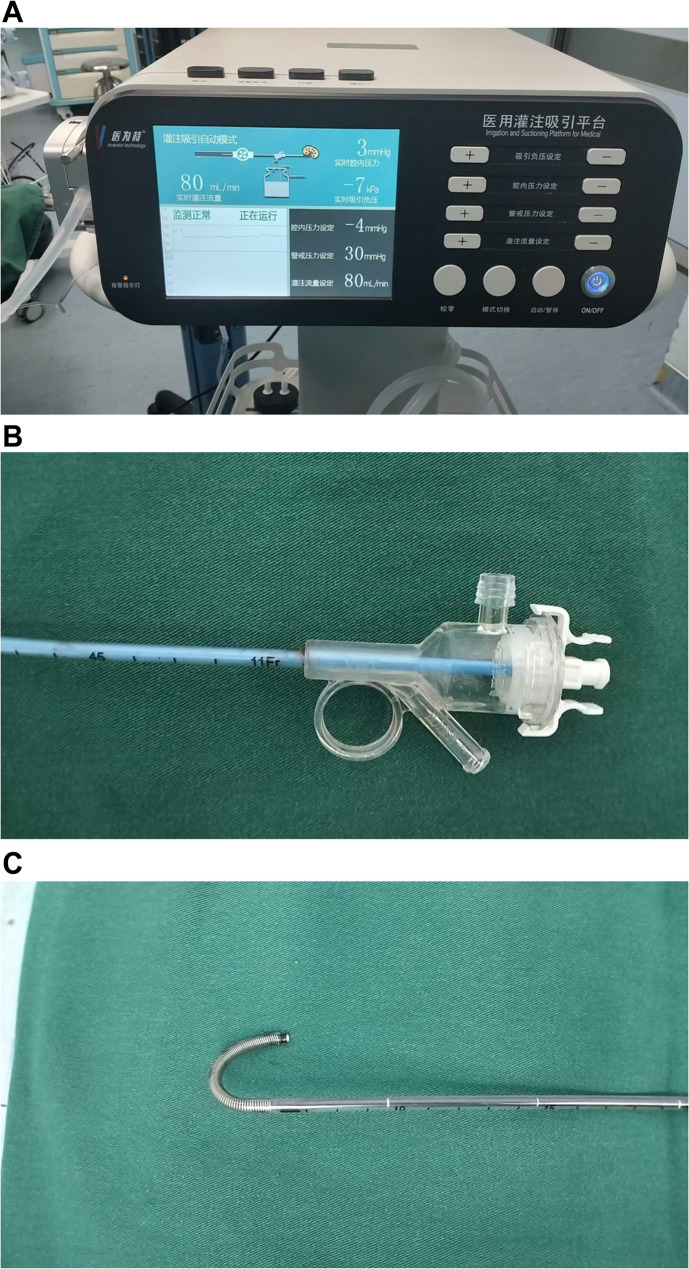
(**a**) Perfusion and suction systems. (**b**) the 3 channels of UAS were devised for flushing, operation of flexible ureteroscopy and suction. (**c**) Tip bendable suction UAS

RIRS with intelligent pressure control is performed with the assistance of medical perfusion and suction systems (Fig. 1A, Inventor Technology, China) and a special ureteral access sheath (UAS), which distributes several holes to measure the renal pelvis pressure at the end of UAS (Fig. 1B and C, Inventor Technology, China). The medical perfusion and suction systems automatically regulate renal pelvic pressure to a protocol-specified value via adjustment of power of suction according to the real-time pressure data monitored by the sensor. The procedure of RIRS with intelligent pressure control was nearly identical to conventional RIRS. However, a key distinction is that, following laser lithotripsy, appropriately sized stone fragments can be aspirated through the medical perfusion and suction systems.

#### Postoperative follow-up and outcome definitions

Operative blood loss was calculated as the difference between preoperative and postoperative hemoglobin levels. The stone-free rate (SFR) was defined as the absence of residual fragments larger than 2 mm, assessed by non-contrast computed tomography (NCCT) with 1 mm slice thickness, performed 30 days postoperatively. We selected the 2 mm threshold because multiple studies have demonstrated that fragments ≤ 2 mm rarely require additional intervention. A systematic review [7] found that residual fragments > 2 mm are associated with a significantly higher risk of stone‑related events and retreatment. Using NCCT at a uniform 30-days’ time point minimizes variability in imaging assessment and selection bias.

### Statistical analyses

Demographic and clinical data were collected from the electronic medical records (EMR) database. Numerical variables with a normal distribution were presented as mean and standard deviation, while those without a normal distribution were presented as median and interquartile range. Categorical variables were described as frequencies and percentages. Statistical analyses were conducted using Student’s t-test for normally distributed numerical variables, the Mann-Whitney *U*-test for non-normally distributed variables, and the chi-square test or Fisher’s exact test for categorical variables. All *p-*values were two-tailed, with *p* < 0.05 considered statistically significant. Factors affecting SFR were analyzed using univariate and multivariate logistic regression All analyses were performed with SPSS 26.0 and R 4.1.0.

## Results

A total of 101 patients were included in the study, with 52 patients undergoing conventional RIRS (Group A) and 49 undergoing RIRS with intelligent pressure control (Group B). There was no statistically differences between Group A and Group B in terms of age (49.8 ± 10.3 vs. 50.6 ± 11.8, *p* = 0.723) and BMI (25.83 ± 2.48 vs. 27.18 ± 5.39, *p* = 0.113). The median stone size for Group A was 15.10 mm (13.47, 17.42) compared to 13.40 mm (11.40, 17.60) for Group B, and the median stone density was 1072.00 HU (994.50, 1164.25) in Group A versus 1049.00 HU (841.00, 1145.00) in Group B, with no statistically significant differences (*p* = 0.066 and *p* = 0.114, respectively). Table 1 provides a summary of all relevant clinical parameters for the patients and stones.

**Table 1:** Demographic and clinical characteristics of patients and stones

Characteristics	Group A^*^(*n* = 52)	Group B^#^(*n* = 49)	*P*
Gender (male/female)	36/16	30/19	0.714
Age (mean ± SD)	49.8 ± 10.3	50.6 ± 11.8	0.723
BMI (mean ± SD)	25.83 ± 2.48	27.18 ± 5.39	0.113
Comorbidities			0.292
Hypertension	15	7	
Diabetes mellitus	14	9	
Ischemic heart disease	0	3	
Stone size (mm), [median (Q1, Q3)]	15.10 (13.47,17.42)	13.40 (11.40,17.60)	0.066
Sone density [median (Q1, Q3)]	1072.00 (994.50, 1164.25)	1049.00 (841.00, 1145.00)	0.114
Stone number (singe/multiple)	32/20	38/11	0.081
Laterality (left/right)	21/31	25/24	0.283
Stone location			0.346
Lower calyx (n, %)	27 (51.9%)	30 (61.2%)	
Other location (n, %)	25 (48.1%)	19 (38.8%)	

* Group A means conventional RIRS

# Group B means RIRS with intelligent pressure control

*BMI* Body Mass Index

Comparative statistics of conventional RIRS and RIRS with intelligent pressure control are shown in Table 2. The SFR of RIRS with intelligent pressure control was significantly higher than for conventional RIRS (89.8% vs. 73.1%, *p* = 0.032). There were not statistically significant differences in operative time (100 min vs. 90 min, *p* = 0.108) or post-operative hospital stays (2 days vs. 2 days, *p* = 0.855) between the two groups. As for post-operative complication, while the incidence of fever was higher in conventional RIRS than in the intelligent pressure control group, this difference was not statistically significant (9.8% vs. 2.0%, *p* = 0.205).

**Table 2:** Comparative statistics of conventional RIRS and RIRS with intelligent pressure control

	Group A		Group B	*P*
Stone-free rate (%)	73.1%		89.8%	0.032
Operative time (min), (Mean ± SD)	100.0 (90.0, 122.5)		90.0 (70.0, 120.0)	0.108
Operative blood loss (g/L), [median (Q1, Q3)]	4.00 (2.00, 6.00)		4.00 (−2.00, 5.00)	0.236
Post-operative hospital stays (days), (Mean ± SD)		2 (2, 3)	2 (1, 3)	0.855
Postoperative complication				0.832
Fever (n, %)	(5, 9.8%)		(1, 2.0%)	0.205
Pain (n, %)	(10, 19.2%)		(10, 20.4%)	0.882
Gross hematuria (n, %)	(2, 3.8%)		(5, 10.2%)	0.387
Other (n, %)	(0, 0%)		(1, 2.0%)	0.485

* Group A means conventional RIRS

# Group B means RIRS with intelligent pressure control

Logistic regression analysis was conducted to explore factors influencing SFR (Table 3). In the univariate logistic regression model, stone size (OR = 1.170, *p* = 0.003), stone density (OR = 1.004, *p* = 0.014), number of stone (OR = 5.684, *p* = 0.001), stone location (OR = 3.571, *p* = 0.035) and surgery procedure (OR = 3.242, *p* = 0.038) was associated with SFR, while BMI (OR = 0.925, *p* = 0.245) did not have a significant effect on SFR. We included the indicators of statistical differences above into the multivariate logistic regression model and found that there was significant correlation between stone size (OR = 1.166, *p* = 0.021), stone density (OR = 1.004, *p* = 0.032), surgery procedure (OR = 4.554, *p* = 0.038) and SFR.

**Table 3:** Uni- and multivariate logistic regression analysis findings for the stone-free rate

Variables	Univariate analysis		Multivariate analysis	
	*P*	OR (95%CI)	*P*	OR (95%CI)
Stone number				
Singe		1.000 (Reference)		1.000 (Reference)
Multiple	0.001	5.684 (1.962 ~ 16.471)	0.105	2.784 (0.806 ~ 9.615)
Stone location				
Other location		1.000 (Reference)		1.000 (Reference)
Lower calyx	0.035	3.571 (1.092 ~ 11.678)	0.148	2.847 (0.689 ~ 11.772)
Surgery manner				
1^*^		1.000 (Reference)		1.000 (Reference)
2^*^	0.038	3.242 (1.069 ~ 9.833)	0.038	4.554 (1.087 ~ 19.076)
Stone size	0.003	1.170 (1.054 ~ 1.299)	0.021	1.166 (1.023 ~ 1.329)
Stone density	0.014	1.004 (1.001 ~ 1.007)	0.032	1.004 (1.000 ~ 1.009)
BMI	0.245	0.925 (0.811 ~ 1.055)		

*OR* Odds Ratio, *CI* Confidence Interval, *BMI* Body Mass Index

1^*^RIRS with intelligent pressure control

2^*^conventional RIRS

## Discussion

Urolithiasis is a prevalent condition worldwide [2]posing a substantial threat to human health and contributing significantly to healthcare costs. A study [8] examining the economic burden of upper urinary tract stone disease in the United States between 2011 and 2018 reported that approximately $10 billion was spent on treatment. While considerable progress has been made in understanding the formation and risk factors of urolithiasis [9]surgical treatment remains a crucial component for managing urinary stones. The choice of surgical approach depends on clinical indications, surgeon preference, and patient discretion [10] [11]. RIRS was introduced to handle intrarenal or proximal ureteric stones less than 20 mm in diameter [11]and there were increasing reports about utilizing RIRS to treat stones>20mm [12]even staghorn stones [13]. However, RIRS can lead to increased renal pelvic pressure, elevating the risk of infection, and its efficacy in clearing stones from the lower calyx is limited. To address these challenges, Song [4] developed an innovative perfusion and suction system combined with a disposable ureteral access sheath equipped with a pressure sensor. This technique, known as RIRS with intelligent pressure control, has been widely adopted in Chinese hospitals, where it has shown promising safety and efficacy outcomes [5] [6]. However, a direct comparison between RIRS with intelligent pressure control and conventional RIRS has not been thoroughly investigated, and there was a lack of reports from China’s primary hospital, which motivated us to conduct this study.

Indeed, the advent of flexible ureteroscopy was relatively early, yet widespread implementation did not commence until the FDA granted approval of digital flexible ureteroscope in 2006 [14]. The technique can be used for lithotripsy or diagnosis and endoscopic treatment of upper urinary tract tumors [15] [16]. However, the early application was restricted partly due to the anatomical complexity of the renal collecting system in certain patients, the risk of infection and the vulnerability of the device. With advancements in medical equipment and surgeon expertise, more and more large stones were managed with RIRS. However, SFR treated with RIRS was not satisfactory [17]. This phenomenon may be attributed to several factors: (a) the limited deflection angle of flexible ureteroscopes, particularly when the laser fiber is in place [18](b) the inability to monitor intrarenal pressure (IRP) in real time, and long duration of surgery leading to elevated IRP, increasing the risk of infection. To address these issues, RIRS devices equipped with intelligent pressure control have been developed. Firstly, the perfusion and suction systems could monitor and stabilize intrarenal pressure within a relatively safe range, which supported to prolong operating time appropriately as needed. Secondly, a bendable-tip suction UAS [19] (Fig. 1C) can improve the deflection angle and reduce the distance between stone fragments and the UAS, which enhaned the efficiency of suction. Overall, these advancements have contributed to improved SFR in RIRS with intelligent pressure control. In our report, SFR was significantly higher with intelligent pressure-controlled RIRS than with conventional RIRS (89.8% vs. 73.1%, *p* = 0.032).

The pathogenesis of infection during RIRS is linked to pyelotubular and pyelovenous backflow due to continuously increasing IRP [20]. Besides, in vivo porcine model, Christina et al. found that increasing renal pelvis pressures during ureteroscopy were associated with increases in irrigation fluid absorption and increases in rate of fluid absorption [21]. As we all know, there were two main methods of irrigation during RIRS including pressured-bag irrigation and hand-operated irrigation pumps. Matthew et al. [22] found that compared to pressured-bag irrigation, hand-operated irrigation pumps obviously increased intrarenal pressure, which induce higher incidence of infectious complications. Anne et al. [23] reported that both solitary and serial manual bolus irrigation cause a sharp increase in intrarenal pressure, especially for the later manner. What’s more, compared to patients without complications, the patients with complications had higher baseline IRP, maximal IRPs and longer duration of elevated pressures, which were all statistically significant (*p* < 0.01). Hence, Anne advised to abandon the maneuver of manual bolus irrigation during RIRS. In conclude, maintaining IRP at a relatively low level was significant to reduce the risk of infection even urosepsis. There were some RIRS devices that influence IRPs, including UAS and automated infusion/pressure control devices [24]. A method to reduce IRP could utilize a larger UAS [25]however, insertion of a larger UAS increased the risk of iatrogenic ureteral injury. As mentioned above, the medical perfusion and suction systems could automatically adjust the renal pelvic pressure to the protocol value via adjustment of suction according to the real-time pressure value monitored by the pressure sensor. The protocol value could be set from − 9mmHg to 30mmHg which ensure that the fluctuation of IRP is within the range. The reason why the upper limit was set at 30mmHg was that the intrarenal flux occurs at pressures of 40 cm H_2_0 (about 30mmHg) [26]. A prospective multi-institutional study measured 120 consecutive adult patients’ intrarenal pressure with pressure guidewire during conventional RIRS, and they found that IRP frequently exceed expected thresholds, of which the highest single pressure peak was 334.2mmHg [27]. Hence, the stability of IRP at a relatively lower level reduced the occurrence of infection. At the same time, compared with conventional RIRS, RIRS with intelligent pressure control could improve the efficiency of stone removal, which not only increased SFR but also shortened the operation time. Song et al. reported the proper size of the stone fragment in ensuring the expulsion during the surgery was ≤ 0.25 mm in traditional UAS and ≤ 0.50 mm in negative-pressure UAS [28]. The short duration of surgery also reduced IRP and the risk of infection. Zhu compared traditional UAS with suctioning UAS during flexible ureteroscopy and found that the incidence of fever (13.9% vs. 5.5%; *p* = 0.009) and urosepsis (6.7% vs. 1.8%, *p* = 0.029) is higher with traditional UAS [29]. However, in our report, although Group A had a higher incidence of fever than Group B, this difference was not statistically significant (9.8% vs. 2.0%, *p* = 0.205), partly owning to the limited sample size, preoperative antibiotic prophylaxis and assessment of preoperative urinalysis and urine culture [30].

There were not statistically significant between RIRS with intelligent pressure and conventional RIRS in terms of operative time, post-operative hospital stay, or postoperative complications. Although we believed that RIRS with intelligent pressure control could improve efficiency of surgery, the lack of an assessment method for actual valid operative time limited our ability to make a precise comparison. Additionally, although the modified UAS distributed pressure sensor at its tip, the diameter was not larger than that of the conventional UAS, which ensured that the operation don’t increase the probability of ureteral injury and therefore don’t exacerbate the patients’ pain and prolong hospitalization.

Our findings indicated that RIRS with intelligent pressure control improved SFR and we aimed to further investigate how the anatomical characteristics of the stones themselves impact SFR. There were multiple scoring systems that describe the anatomical features of stones based on various dimensions and evaluate factors affecting SFR after RIRS, including Resorlu-Unsal Stone score (RUSS), R.I.R.S. scoring system score, Modified Seoul National University Renal Stone Complexity (S-ReSC) score, S.T.O.N.E. score, Ito’s nomogram, T.O. HO. score and so on [31] [32]. Admittedly, these scoring systems were elaborately devised and well validated, however, there were controversial conclusions during external validation [33] [34]. The majority of systems concentrated on four key factors: stone size, stone density, the number of stones, and stone location, while some also accounted for stone volume and hydronephrosis for a more comprehensive assessment. Stone volume can be calculated using methods such as the ellipsoid formula or 3D reconstruction, but these approaches have not proven significantly more accurate than measuring stone size alone [35] [36]. According to our experience, we believed that the effect of hydronephrosis on SFR was dichotomous, which meant severe hydronephrosis indeed pose difficulties in the treatment of stones. Batuhan et al. [37] also found that the presence of preoperative hydronephrosis did not decrease the success of flexible ureteroscopy. Shunsuke Hori et al. [38] reported that stone size, stone density, and the location of the stones were independent risk factors of SFR during flexible ureteroscopy in multiple logistic regression. Based on this, they established T.O. HO. score, and some reports^39 40^ had externally validated the accuracy of the scoring system successfully. In our report, we found the analogous results indicating that stone size, stone density, number of stones, and location of stones were associated with SFR of RIRS in univariate logistic regression. When the above parameters were included in the multiple logistic regression model, we found that stone size and density could affect SFR during RIRS. Furthermore, we observed that, in both univariate and multivariate models, RIRS with intelligent pressure control significantly improved SFR compared with conventional RIRS.

## Limitations

There were several limitations in our report. First, the sample size was small, which may influence the outcomes. The limited sample size could induce to a Type II error, which could reduce statistical power. Second, all the patients were selected from one center, which may result in selection bias. Addtionally, the choice of the surgery was based on equipment availability and surgeon preference rather than randomization, which could also induce to selection bias. However, there is currently a lack of relevant reports comparing conventional RIRS with RIRS with intelligent pressure control at the present. Besides, we evaluated the relevant factors affecting SFR, and further validated the validity of RIRS with intelligent pressure control, which was also lacking in other relevant reports.

## Conclusion

In summary, compared to conventional RIRS, RIRS with intelligent pressure control significantly stabilizes IRP at a relatively safe level and improves the stone-free rate without increasing complications, prolonging hospitalization, or extending surgical time. Further multicenter studies with larger sample sizes are warranted to confirm the clinical value of this promising method

## Supplementary Information

Supplementary Material 1.

## Data Availability

Availability of data and materials The datasets used and/or analyzed during the current study are available from the corresponding author on reasonable request.

## Abbreviations

RIRS: Retrograde intrarenal surgery
SFR: Stone free rate
ESWL: Extracorporeal shockwave lithotripsy
URS: Ureteroscopy
PCNL: Percutaneous nephrolithotomy
UAS: Uretral access sheath
IRP: Intrarenal pressure
