# Supplementary material for: Retrograde intrarenal surgery with intelligent pressure control: experience from a primary hospital in China
Source: BMC Urol. 2025 Aug 26;25:218. doi: 10.1186/s12894-025-01910-8 (PMC12379481; doi:10.1186/s12894-025-01910-8)

Figure 2A Variables affecting SFR in univariate logistic regression


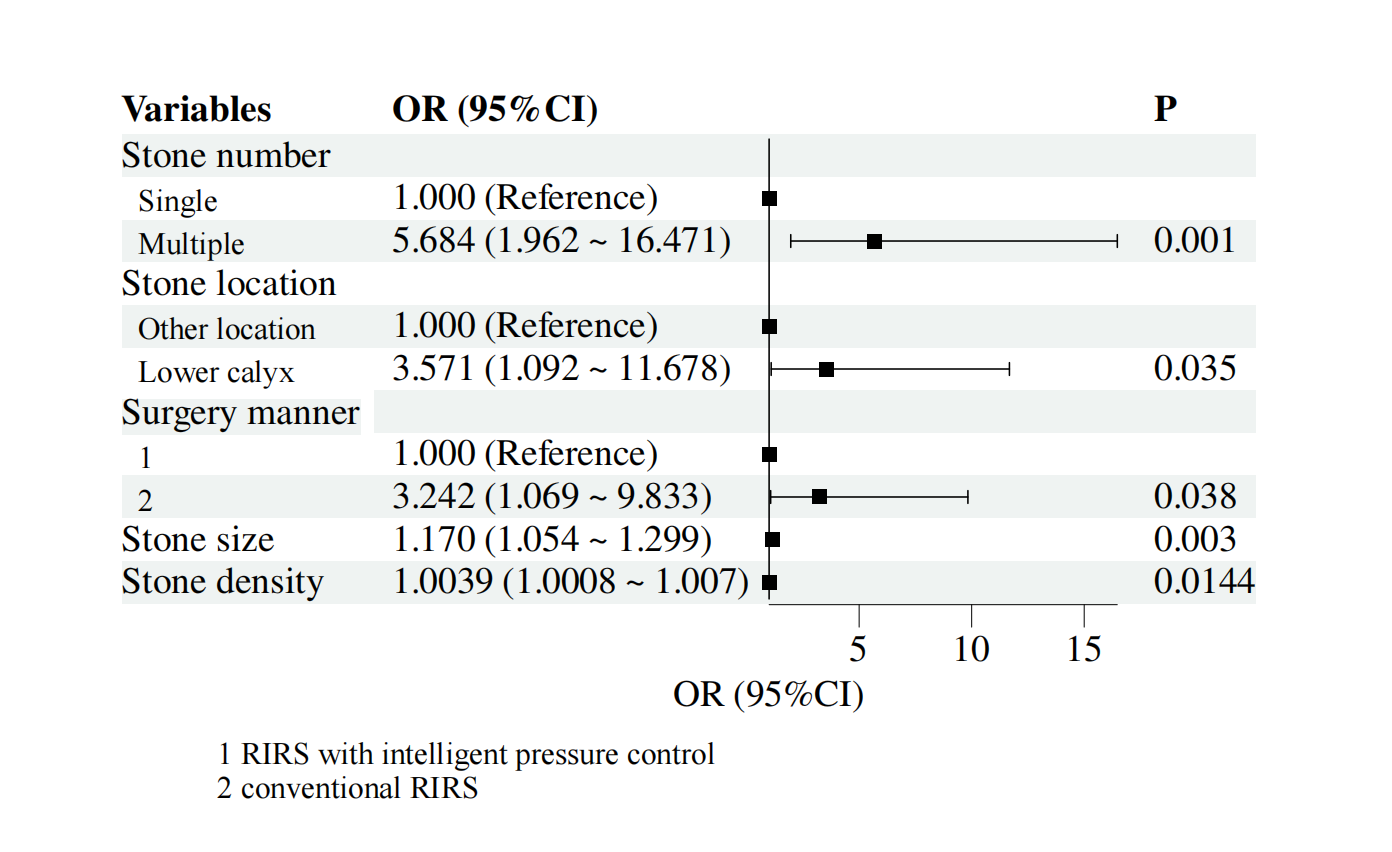


Figure 2B Variables affecting SFR in multivariate logistic regression


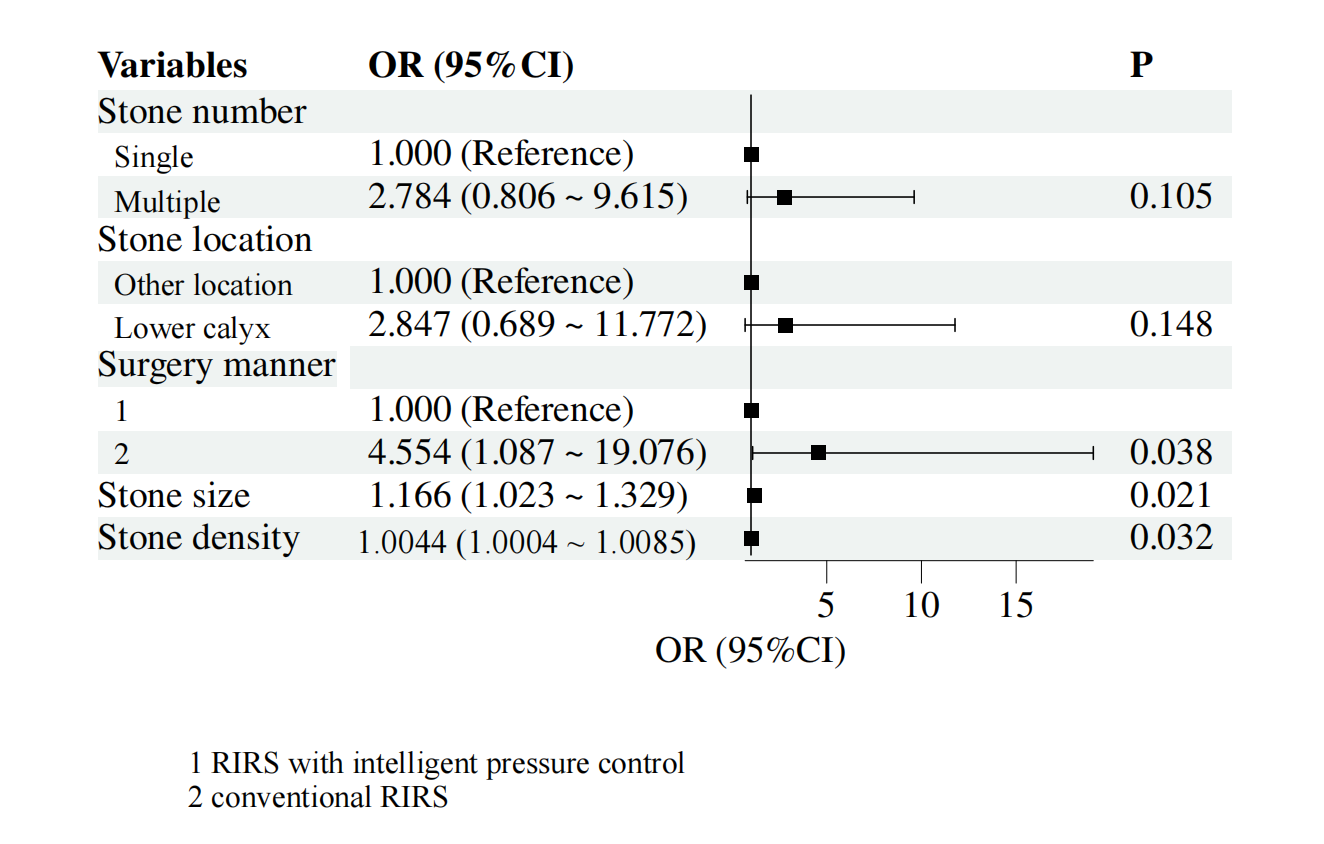

Supplement: Supplementary file 1 — Supplementary Material 1. [file 12894_2025_1910_MOESM1_ESM.docx]
